# LoRaWANSim: A Flexible Simulator for LoRaWAN Networks

**DOI:** 10.3390/s21030695

**Published:** 2021-01-20

**Authors:** Riccardo Marini, Konstantin Mikhaylov, Gianni Pasolini, Chiara Buratti

**Affiliations:** 1 Wilab, CNIT and DEI Department, University of Bologna, Viale Risorgimento 2, 40136 Bologna, Italy; r.marini@unibo.it (R.M.); gianni.pasolini@unibo.it (G.P.); 2 Centre for Wireless Communications, University of Oulu, Erkki Koiso-Kanttilan katu 3, 90570 Oulu, Finland; konstantin.mikhaylov@oulu.fi

**Keywords:** LPWAN, LoRaWAN, LoRa, internet of things, network simulator, MATLAB, simulation, performance, model, analysis

## Abstract

Among the low power wide area network communication protocols for large scale Internet of Things, LoRaWAN is considered one of the most promising, owing to its flexibility and energy-saving capabilities. For these reasons, during recent years, the scientific community has invested efforts into assessing the fundamental performance limits and understanding the trade-offs between the parameters and performance of LoRaWAN communication for different application scenarios. However, this task cannot be effectively accomplished utilizing only analytical methods, and precise network simulators are needed. To that end, this paper presents LoRaWANSim, a LoRaWAN simulator implemented in MATLAB, developed to characterize the behavior of LoRaWAN networks, accounting for physical, medium access control and network aspects. In particular, since many simulators described in the literature are deployed for specific research purposes, they are usually oversimplified and hold a number of assumptions affecting the accuracy of their results. In contrast, our simulator has been developed for the sake of completeness and it is oriented towards an accurate representation of the LoRaWAN at the different layers. After a detailed description of the simulator, we report a validation of the simulator itself and we then conclude by presenting some results of its use revealing notable and non-intuitive trade-offs present in LoRaWAN. Such simulator will be made available via open access to the research community.

## 1. Introduction

The Internet of Things (IoT) represents an ecosystem of autonomous devices able to sense, communicate and provide actuation without the need for human intervention. This technology is already adopted in many scenarios, from industry to agriculture, and from wildlife to smart cities [1], and the number of IoT devices is expected to grow to 22 billion by 2025 [2]. Focusing the attention, in particular, on large-scale IoT networks, long-range connectivity and energy efficiency are challenging requirements that have been met by low power wide area network (LPWAN) technologies, such as LoRaWAN, NB-IoT [3] and SigFox [4]. Such networks combine long battery life (even ten years) and wide coverage (even several kms), at the cost of low bit rate.

Among the LPWAN technologies, LoRaWAN is deemed as one of the most promising, being relatively flexible and straightforward, both technology-wise and business-wise [5,6]. In a LoRaWAN network, end devices (EDs), representing IoT nodes (e.g., sensors or actuators), send packets to gateways (GWs), which forward them to a network server (NS) they are connected to via Internet (using WiFi, 3G/4G, Ethernet, etc.). The NS serves as a centralized entity responsible for the network’s upkeep.

In the physical layer, LoRaWAN relies on LoRa, which is a proprietary physical layer solution patented by Semtech Corporation (https://www.semtech.com/lora), and the medium access control (MAC) and network layers have been defined by the LoRa Alliance (https://lora-alliance.org/) in their respective specifications [7]. The LoRa modulation is based on the chirp spread spectrum, which exploits chirps whose frequencies increase or decrease linearly over a certain amount of time. One of the most important parameters of the physical layer is the spreading factor (SF) (see Section 3.1 for further details), which increases for increasing values of the ratio between the signal bandwidth and the symbol rate. While keeping the bandwidth constant, it is possible to improve the receiver sensitivity (and thus the maximum communication distance) by increasing the time on air (ToA) (duration of a packet transmission), that is, by increasing the SF. Transmissions using different spreading factors are often assumed to be orthogonal, even though perfect orthogonality is not reached [8,9].

LoRa devices mainly operate in license-free Industrial, Scientific and Medical (ISM) bands, whose availability and usage conditions somewhat differ for various regions of the globe [10]. Specifically, in the EU, the most widespread implementation is based on the 863–870 MHz ISM band, even though a version of LoRa working at 2.4 GHz is gaining much attention. This paper considers the operation on the 863–870 MHz band, which is subject to duty cycle (DC) constraints (see [10]).

Given the LoRaWAN potential, the research community has been dedicating significant efforts to its study, both theoretically and experimentally [11]. A number of open-source network simulators have been developed over the recent years [12]; however, most of them have been created for validating one specific research target. Therefore, they tend to be focused on a specific aspect and are often limited by a number of assumptions and cannot properly capture the operation of the LoRaWAN protocol stack as a whole. Another somewhat limiting factor is the steep learning curve required to use these tools, which is due to the use of highly effective, but rather complex discrete-event simulation frameworks.

One of the most popular LoRaWAN simulators available today is LoRaSim [13], a discrete-event simulator realized using SimPy. It can emulate a network of devices and gateways randomly positioned on a 2-dimensional grid and implements a channel model based on the well-known log-distance path loss. Two performance metrics are provided as outputs: data extraction rate and network energy consumption, which refer to the overall network and not to the behavior of the individual ED. LoRaSim, though, lacks a number of features, such as accounting for the imperfect SF orthogonality, downlink traffic and DC limitation. A closed-source expansion of LoRaSim is presented in [14]. The main improvement concerns bidirectional communication, but the other features are still missing.

An ns-3 (ns-3 is a discrete-event network simulator for Internet systems, targeted primarily for research and educational use. ns-3 is free software, licensed under the GNU GPLv2 license, and is publicly available for research, development, and use [15]) based simulator implementing class A is presented in [16], and it has been extended by the authors themselves to include also downlink traffic [17]. However, there is no characterization of the energy performance.

LoRaFREE [18] implements downlink communications and spreading factor (SF) imperfect orthogonality in a network with one full-duplex GW and EDs generating periodic traffic only; in addition, the channel is based on the log-distance path loss model. LoRaEnergySim [19], instead, is a tool based on the WiMOD iC880A GW, which focuses on the energy consumption, even though it still assumes perfect SF orthogonality.

The rest of the paper is organized as follows: Section 2 outlines the novel contributions of the simulator proposed in this paper; Section 3 presents the background of LoRa and LoRaWAN protocols; Section 4 describes the implementation of the simulator, with particular emphasis on the scenario and the transmission mechanism, and the results it is able to produce; Section 6 and Section 7 show the results offered by the simulator and a comparison with some analytical models and experimental outcomes; finally, Section 8 summarizes the results and the key takeaways.

## 2. Original Contributions

The original contributions of this paper are twofold. First, we describe the architecture of the LoRaWAN simulator, which we release as open access; it integrates both a MAC layer and physical layer functionalities. Second, we provide original results obtained by means of the simulator, which give insights into the performance of LoRaWAN and the impacts of different setups (e.g., in terms of coding rate).

As for the simulator itself, the novelty of our approach lies in the integration of two separate simulators, namely, the MAC layer simulator and the physical layer simulator, which interact with each other at run-time. In particular, the physical layer simulator is in charge of providing the outcome (success/failure) of each single transmission to the MAC layer simulator, which knows who is transmitting to whom and when. This depends on a number of aspects and parameters (e.g., experienced signal-to-noise ratio, adopted SF and coding rate, interleaving, gray coding, modulation/demodulation) that are accurately considered/reproduced at the physical layer. Said mechanism will be addressed more precisely in Section 4. This level of integration is not usual in currently available LoRaWAN simulators, or in network simulators in general. Among the other unique features, which, to the best of our knowledge, are not present in the state-of-the-art tools are: (i) support of multiple gateways; (ii) the possibility of prioritizing and enabling/disabling the receive windows; (iii) accounting for uplink–downlink interference in RX1, and modeling half or full-duplex LoRaWAN gateways.

As for the numerical results, several original results are provided. Specifically, we investigate the impacts of different coding rates in interference-limited and noise-limited scenarios, also showing that in heavily interference limited conditions, the adoption of powerful coding rates is counterproductive for both the delivery rate and the energy consumption. Moreover, we assess the impact of downlink transmissions (e.g., acknowledgments) on the average energy consumption of EDs, also showing that increasing the number of gateways affects not only the packet delivery rates in uplink and downlink, but also the average consumption of devices, and hence, ultimately, the battery life.

## 3. Technology Background: LoRa and LoRaWAN

In this section, the fundamentals of LoRa and LoRaWAN relevant to understanding the key aspects of the protocol and architecture are presented and discussed.

### 3.1. LoRa PHY

At the physical (PHY) layer, LoRa adopts the *M*-ary chirp spread spectrum (CSS) modulation, which is based on chirps, that is, sine-wave signals whose frequencies sweep linearly with time. Denoting with $f_{0}$ the central frequency of the sweep interval $\left[f_{0}-\frac{BW}{2},f_{0}+\frac{BW}{2}\right]$, and assuming t=0 as the signal starting instant, a single LoRa chirp can be mathematically expressed as

(1) $$c\left(t\right)=V_{0}\cos\left(2\pi f_{0}t+2\pi \int_{0}^{t}\Delta f\left(s,\xi \right)d\xi +\phi_{0}\right),\;\;0\leq t\leq T_{s}$$

where

- $V_{0}>0$ is the chirp amplitude;
- s∈{0,⋯,M−1} is the modulation symbol;
- Δf(s,t) is the symbol-dependent instantaneous frequency-offset, ranging in the interval $\left[-\frac{BW}{2},\frac{BW}{2}\right]$;
- $\phi_{0}$ is the signal phase at the initial instant t=0;
- $T_{s}$ is the chirp duration.

In particular, LoRa uses *M* differently-shaped chirps, each of which is in one-to-one correspondence with the *M* symbols of the modulation alphabet S={0,⋯,M−1}: Given a modulation symbol s∈S, the corresponding Δf(s,t) linearly increases starting from $-\frac{BW}{2}$. Then, when the maximum frequency-offset $\frac{BW}{2}$ is reached, Δf(s,t) wraps around to $-\frac{BW}{2}$ and keeps on increasing linearly until $\Delta f\left(s,t=T_{s}\right)=\Delta f\left(s,0\right)$. $T_{s}$, which represents the chirp duration, is usually referred to as *symbol interval*.

According to the LoRa PHY-layer specification, the modulation parameters are chosen such that

- BW∈{125,250,500} kHz;
- $M=2^{SF}$, with SF denoting the previously introduced spreading factor;
- SF ∈{7,8,9,10,11,12};
- $T_{s}BW=M$.

By operating with high SF values, LoRa transmitters increase their communication ranges [20]; robustness against channel impairments and interference from third systems, along with frequency selectivity and the Doppler effect, are also enhanced by increasing SFs. On the other hand, this results in a low data rate and long ToA, which are the main drawbacks of operating with high SFs.

The physical layer bit rate $R_{b}$ depends on SF, the sweep interval BW and the coding rate CR of the forward error correction (FEC) mechanism (which encodes 4 data bits into codewords of 5–8 bits for CR∈[1,..,4], respectively) and is given by [21]:

(2) $$R_{b}=SF\cdot \frac{\frac{4}{4+CR}}{\frac{2^{SF}}{BW}}\;\left[\mathrm{bit}/s\right].$$

The indicative values (denoting the maximum instantaneous data rate) are provided in Table 1 assuming, as an example case, BW = 125 kHz and CR = 1.

The bit rate has a direct impact on the ToA of transmissions, which is the time needed to transmit a packet on the wireless channel, and it is computed [22] with the following equations:

(3) $$T_{\mathrm{symbol}}\left(SF\right)=\frac{2^{SF}}{BW}$$

(4) $$T_{\mathrm{preamble}}\left(SF\right)=\left(L_{\mathrm{preamble}}+4.25\right)\cdot T_{\mathrm{symbol}}\left(SF\right)$$

(5) $$L_{\mathrm{payload}}=8+\left\lceil \frac{\left(8B-4SF+28+16-20H\right)}{\left(4SF\right)}\right\rceil \cdot \left(CR+4\right)$$

(6) $$T_{\mathrm{payload}}\left(SF\right)=L_{\mathrm{payload}}\cdot T_{\mathrm{symbol}}\left(SF\right)$$

(7) $$ToA\left(SF\right)=T_{\mathrm{preamble}}\left(SF\right)+T_{\mathrm{payload}}\left(SF\right)\;\left[s\right]$$

where *B* is the payload size in bytes; H=0 when the header is enabled and H=1 when no header is present; $L_{\mathrm{preamble}}$ and $L_{\mathrm{payload}}$ are the lengths in symbols of the preamble and the payload respectively.

### 3.2. LoRaWAN

**Figure 1:**
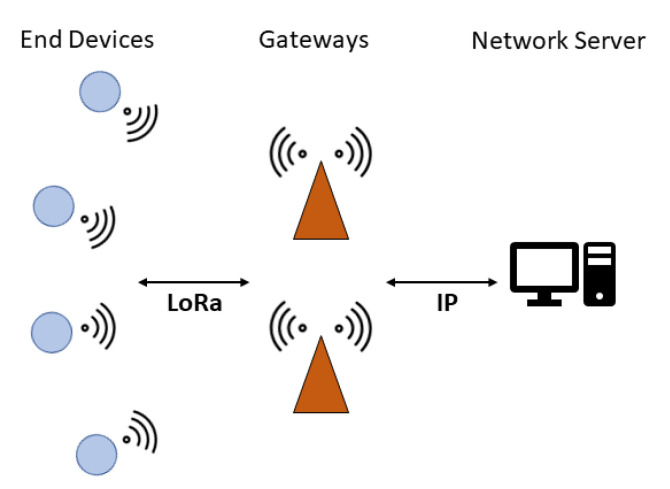
LoRaWAN network architecture.

On top of the LoRa PHY, the LoRaWAN protocol builds up the upper protocol layers. Communication between EDs and GWs is implemented via LoRa, whereas GWs are connected via standard Internet Protocol (IP) connections, such as Wi-Fi, Ethernet or 3G/4G, to the NS, resulting in a star-of-stars topology (Figure 1). EDs are not associated with a specific GW but rather to a NS, and all GW data received from an ED forward them to the NS, which is then in charge of discarding duplicates, sending acknowledgements (ACKs) and managing the overall network.

The access to the radio channel is ALOHA-based complemented by selecting one of the available frequency channels, so an ED sends a packet whenever it has data to send. By standard, the network channels can be freely attributed by the network operator. However, in the EU868MHz band, three default channels must be implemented in every end-device [10]. Those channels are the minimum set that all network gateways should always be listening on. They are reported in Table 2. In addition to these, extra channels (up to 16 in total) can be optionally configured.

LoRaWAN defines three classes of devices, which differ primarily with respect to the support of the downlink communication capabilities and which are labelled A, B and C.

**Figure 2:**
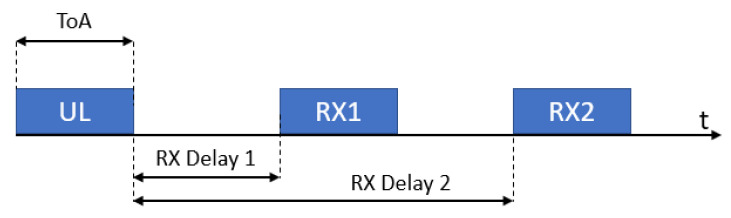
RX Windows in Class A.

- Class A devices’ uplink transmission is followed by up to two short downlink receive slots (denoted receive window 1 (RX1) and receive window 2 (RX2), respectively) after two different fixed RECEIVE_WINDOW_DELAY intervals (the standard suggests using 1 s delay between the end of an uplink and RX1, and 2 s delay between the end of an uplink and RX2), as can be seen in Figure 2. Class A is primarily intended for EDs with limited energy availability (e.g., battery-powered ones), such as sensors, since it results in the lowest power consumption.
- Class B devices allow for more than two receive slots, opening extra receive windows at scheduled times. Synchronization between EDs and GWs is kept via periodic beacons broadcast by a GWs.
- Class C devices have a nearly continuously open receive window except for the time spent in uplink transmissions, so they are always reachable by the network, which is attained at the cost of an increased power consumption.

Furthermore, two transmission modes, which can be selected on a per-packet basis, are defined by the LoRaWAN specifications:

- Confirmed mode: When an ED sends an uplink packet, it expects to receive an ACK packet from the NS (through the GW) after its transmission, and it may continue transmitting the same message if no ACK is received.
- Unconfirmed mode: No ACK is sent by the NS, so the ED does not know if the packet has been correctly received.

The NS is also in charge of controlling the transmission parameters (SF and transmit power $P_{T}$) of the EDs when the adaptive data rate (ADR) algorithm is active. The ADR algorithm is specifically designed to increase the data rate and reduce the energy consumed by the ED. It is not specified in the standard; however, Semtech provides a recommendation in [23] and most of NSs, to the best of our knowledge, operate based on it; its performance has been evaluated in [24]. Note that ADR should be used only if an ED operates in sufficiently stable RF conditions (e.g., static devices with fixed locations). As a matter of fact, the ED itself is in charge of deciding when the ADR should be active, so it should be able to understand whether it is in stable conditions on its own. If this is the case, the ADR bit in the frame header of the packet should be set to 1, informing the NS that the ED is ready to receive ADR commands. When it is set, the NS starts collecting information about the uplink transmissions by the device, which contain the frame counter, signal-to-noise ratio (SNR) measurements and the number of GWs that received each uplink frame. Then, after 20 transmissions, the algorithm will go through a number of iterations based on the maximum SNR measured among these transmissions, and at the end, it will provide the SF and the $P_{T}$ the device should use starting from the next transmission. This information will be sent to the device via a *LinkADRReq* command, which should be acknowledged with a *LinkADRAns*. Algorithm 1 shows the implementation of ADR at the NS.
**Algorithm 1:** NS ADR Algorithm for a given ED. **Input**: SF, $P_{T}$, $SF_{\min}=7$, Margin = 10 dB $SNR_{\max}=max\left\{last\;20\;uplink\;packets\;received\right\}$, $SNR_{\min}$ given in Table 3 by setting SF, $SNR_{\mathrm{margin}}=SNR_{\max}-SNR_{\min}-Margin$, $N_{\mathrm{step}}=\left\lfloor \frac{SNR_{\mathrm{margin}}}{3}\right\rfloor$ **Output**: SF, $P_{T}$1 **if**
$N_{\mathrm{step}}>0$
**then**(2  **while**
*$N_{\mathrm{step}}>0\;\&\;SF>SF_{\min}$*
**do**3   (SF=SF−14   $N_{\mathrm{step}}=N_{\mathrm{step}}-1$5  **while**
*$N_{\mathrm{step}}>0\;\&\;P_{T}>P_{T_{\min}}$*
**do**6   ($P_{T}=P_{T}-3\;dB$7   $N_{\mathrm{step}}=N_{\mathrm{step}}-1$8 **else**(9  **while**
*$N_{\mathrm{step}}<0\;\&\;P_{T_{\mathrm{temp}}}<P_{T_{\max}}$*
**do**10   ($P_{T}=P_{T}+3\;dB$11   $N_{\mathrm{step}}=N_{\mathrm{step}}+1$12 **return**
SF, $P_{T}$

## 4. LoRaWAN Simulator Description

**Figure 3:**
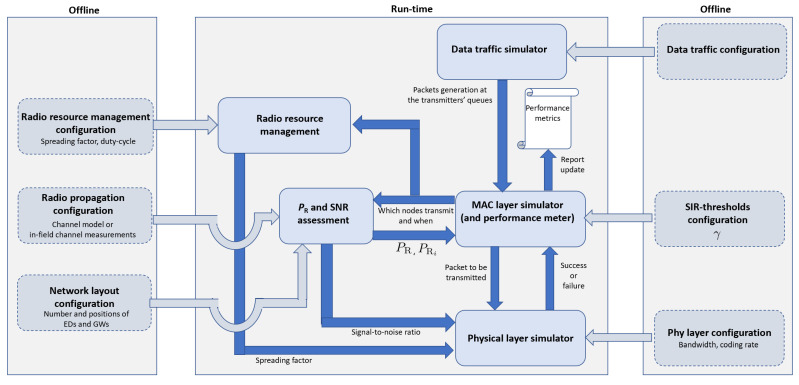
Simulator block scheme.

This section details the key features of the LoRaWAN simulator which is available free from https://github.com/kvmikhayl/LoRaWAN_simulator. As shown in the block scheme illustrated in Figure 3, it implements both PHY and MAC layer functionalities.

The PHY-layer simulator implements a complete LoRa transceiver (transmitter + receiver), thereby generating the modulated signal and performing the demodulation tasks, whereas the MAC layer simulator manages the channel multiple access, thereby implementing the data traffic (who transmits to whom and when), accounting for mutual interference and the presence of multiple gateways. Specifically, the simulator considers class A LoRaWAN devices (support of class C is also available) operating in the EU 863–870 MHz ISM band, and supports a number of features, including both uplink and downlink communications, imperfect SF orthogonality, capture effect, full/half-duplex GW operation, uplink-downlink interference, DC limitations and energy consumption estimation.

The simulation tool is extremely flexible, as it allows one to tune a large number of parameters, related to the PHY layer, the LoRaWAN protocol and the network itself. The outcomes are provided in terms of overall delivery rate, for both uplink and downlink, and energy consumption values for the individual devices and the network as whole.

In the following, the function of each block shown in Figure 3 is discussed.

### 4.1. Offline Configuration Operations

Prior to the simulation execution, a configuration step must be carried out offline, which consists of the definition of the scenario (e.g., the network layout) and of a number of parameters, which rule the network behavior. This step is discussed hereafter.

**Network layout configuration.** For each simulation, an area of interest is defined by the user (by default, a circular shape is assumed), in which *N* EDs and *G* GWs are deployed either manually (e.g., where they are actually located in a real deployment), or automatically, in fixed (e.g., on a grid) or in random positions.

**Radio propagation configuration.** An Okumura–Hata channel model [25] is implemented as the default model in the simulator, even though other channel models can be defined by the user. The power received by a GW, $P_{R}$, is computed as a function of the transmit power, $P_{T}$, as $P_{R}\left[\mathrm{dBm}\right]=P_{T}\left[\mathrm{dBm}\right]+G_{A}^{T}\left[\mathrm{dB}\right]+G_{A}^{R}\left[\mathrm{dB}\right]-L\left[\mathrm{dB}\right]$, where $G_{A}^{T}$ is the transmitting antenna gain; $G_{A}^{R}$ is the receiving antenna gain; *L* represents the path loss in dB,

(8) $$\begin{array}{cc} L= & 69.55+26.16\;log_{10}\left(f\right)-13.82\;log_{10}\left(h_{b}\right)-C_{H}+\\ \; & \left(44.9-6.55\;log_{10}\left(h_{b}\right)\right)\cdot log_{10}\left(d\right)+s \end{array}$$

where *f* is the frequency in MHz, $h_{b}$ is the height of the GW in m, *d* is the distance between the transmitter and the receiver in km and $C_{H}$ is the antenna height correction factor, which varies according to the frequency and the size of the city,

(9) $$C_{H}=3.2{\left(log_{10}\left(11.75\;h_{m}\right)\right)}^{2}-4.97$$

with $h_{m}$ denoting the height of the ED antenna in m. It should be highlighted that (9) is valid for large urban areas and f>400 MHz. Finally, *s* represents random channel fluctuations due to shadowing, modeled as a Gaussian random variable, with zero mean and standard deviation σ; that is, $s∼N(0,\;\sigma^{2})$. The user can choose the transmit power $P_{T}$, σ and the height of the EDs/GWs.

Note that the discussed layout and propagation models can be defined by the user manually based, e.g., on empirical measurements. The respective functionality is supported by the simulator.

**Radio resource management configuration.** The policy adopted for the choice of the SF can be defined by the user, along with the DC configuration. As for the former, the user can dictate a specific SF for each ED or let the simulator make a decision based on the ADR. With reference to the DC, instead, the DC constraints can be disabled or enabled; in the latter case, these are automatically satisfied according to the regional limitation reported in [10]. In particular, DC limitations are specified according to LoRaWAN specification version 1.0.1, which dictates that a device (i.e., ED or GW), after sending a packet of duration ToA seconds, must not use the same frequency sub-band for the next $\mathrm{ToA}(\frac{1}{\mathrm{DC}}-1)$ seconds. In addition, the user can also define the transmission parameters related to both RX windows. For RX1, the RX1DROffset parameter, which is the difference between the SF used in uplink and in RX1, is chosen by default according to Table 4 (the user can introduce a different table, if needed). For RX2, instead, SF is fixed to 12 by the standard, even though the user can decide to change such parameter.

**Data traffic configuration.** The traffic generation models, for both the uplink and the downlink, are also configured by the user, along with the size of uplink and downlink packets in bytes ($B_{UL}$ and $B_{DL}$, respectively), the presence of the packet header *H* and the length of the preamble $L_{preamble}$. When the default configuration is adopted, data packets are generated by EDs periodically every *T* seconds. The user can also define the probability of generating a downlink message after an uplink packet; by default, this is set to 1.

**SIR threshold configuration.** The MAC layer simulator is in charge of reproducing the ALOHA-based access protocol adopted by LoRaWAN, which does not depend on parameters to be configured. However, within the simulator this block also evaluates the signal-to-interference ratio (SIR) experienced by a receiver due to simultaneous transmissions, in order to check whether the packet is correctly received or not. As detailed in Section 5, the SIR is compared with a threshold γ, dubbed *SIR-threshold*, which can be defined by the user. Clearly, evaluating the success/failure of a transmission in the presence of interference is by no means a MAC layer task; in the simulator perspective, however, the MAC layer simulator block is the most appropriate for the SIR assessment, because only this block knows which nodes (if any) are simultaneously transmitting hence might interfere each other.

**Physical layer configuration.** The bandwidth BW of the modulated signal is a user-defined parameter, along with the coding rate CR of the forward-error-correcting code adopted to reveal/correct transmission errors. Indeed, the encoding process should be carried out at the logical link control (LLC) sub-layer but, for the sake of simplicity, in our simulator it is carried out by the PHY-layer simulator. Note that in the current LoRaWAN specification both BW and CR are pre-specified for each region. Our models allow one to update these parameters, thereby adapting the simulator to other regions and/or novel modulation-coding schemes.

Table 5 summarizes the key parameters a user can define.

### 4.2. Run-Time Operations

During the execution, the data traffic simulator, which oversees the whole network, provides the MAC layer simulator with the time instants when data packets, generated either by EDs or GWs, enter the respective transmission queues. The MAC layer simulator, in turn, reproduces the behavior of the ALOHA channel access protocol for all devices in the network, thereby deciding which nodes, among those with queued packets, transmit and when.

This information is passed to the SNR assessment block that, given the positions of the transmitting nodes and the adopted channel model/channel measurements, derives the SNR experienced by receivers. Such information, along with the current SF value provided by the radio resource management, is used by the physical layer simulator, which generates the modulated signal corrupted by noise and demodulates it in order to assess if the packet is correctly received or not. Clearly, all accompanying operations, such as interleaving/de-interleaving, gray coding/decoding and channel coding/decoding, are carried out as well. The transmission outcome, either success or failure, is passed to the MAC layer simulator for any consequent action. The MAC layer simulator also gets from the SNR assessment block the values of the useful and interfering power at the receiver under investigation. This information is used to assess whether the interference level is such to prevent the correct reception. As detailed in Section 5, this assessment is carried out by comparing the SIR experienced by the receiver under investigation and the SIR-threshold γ. Given the result of such comparison and the success/failure outcomes of the physical layer simulator (which does not account for interference), the MAC layer simulator updates the performance counters, which are then used to compute the final performance metrics.

## 5. Performance Metrics

At the end of the simulation, three default performance metrics are provided, which are discussed hereafter.

### 5.1. Performance Metrics: Uplink Delivery Rate

The delivery rate provided by the simulator refers to the packets received by GWs. Two impairments are taken into account that might prevent GWs from correctly receiving transmitted packets, namely, noise and interference.

As mentioned above, the LoRa PHY simulator implements the whole transmitter → AWGN channel → receiver chain. In particular, the PHY-layer simulator reproduces the operations carried out by the transmitter (channel coding, interleaving, gray coding and modulation), the addition of AWGN noise in the channel and the receiver behavior (demodulation, deinterleaving, decoding). Thus, given the frame of data bits to be transmitted, the adopted SF, CR and BW and the actual SNR that characterizes a given link, the PHY-layer simulator assesses whether the currently transmitted packet is correctly received or not.

The possible presence of interference is also considered, taking into account the possibility that, even though a collision happens between two LoRa packets (considering both uplink and downlink), one of them could be correctly received if one of the signals is strong enough. In this case, the simulator supports the capture effect mechanism, so the packet is correctly received provided that the SIR that is the ratio between the useful received power and the sum of the interfering powers is above a given threshold, γ:

(10) $$SIR=P_{R}/\sum_{i}P_{R_{i}}\geq \gamma$$

where $P_{R}$ is the received power of the target ED, $P_{R_{i}}$ is the received power of the i-th interfering signal and γ depends on the SF used, as specified by a Table 6 [9] (the table can also be modified by a user, if desired). In particular, since we account for inter-SF interference, the inequality should be verified for all the interfering signals by summing the received power for each SF.

Condition (10) is used by the MAC layer simulator to decide whether the reception of a packet by GWs is prevented by the interference. In addition, since our simulator supports both full-duplex and half-duplex GWs, interference between transmissions takes into account also the potential uplink/downlink interference and the very possibility of the GW to transmit and receive packets simultaneously. The type of the GWs is specified as a part of network pre-configuration.

### 5.2. Performance Metrics: Energy Consumption

The simulator is able to estimate the LoRaWAN energy-performance by computing the energy consumed by each ED. We assume an ED working in class A and transmitting an uplink packet, followed, optionally, by the reception of a downlink packet. Therefore, the simulator takes into account the energy spent in: uplink transmission, RX delay 1, downlink reception in RX1, RX delay 2, downlink reception in RX2 and sleep until the next uplink transmission, as reported in (11).

(11) $$\begin{array}{cc} E= & E_{UL}+E_{RX_{Delay1}}+E_{DL_{RX1}}+E_{RX_{Delay2}}+E_{DL_{RX2}}+E_{Sleep}=\\ \; & V\;I_{TX}\;T_{TX}+V\;I_{RX_{Delay1}}\;T_{RX_{Delay1}}+V\;I_{DL_{RX1}}\;T_{DL_{RX1}}+\\ \; & V\;I_{RX_{Delay2}}\;T_{RX_{Delay2}}+V\;I_{DL_{RX2}}\;T_{DL_{RX2}}+V\;I_{Sleep}+T_{Sleep} \end{array}$$

In (11), *V* represents the voltage, *I* is the current and *T* is the time spent in each state. In particular, the time passed in transmission and reception, $T_{TX}$ and $T_{RX}$ (in case a downlink packet has been sent), are considered equal to the ToA of the packet, which is computed from (7). The time intervals spent in $T_{RX_{Delay1}}$ and $T_{RX_{Delay2}}$ are defined by the standard, as reported in Section 3. If a downlink packet has not been sent or the packet has not been correctly received due to interference, the time intervals spent in reception during RX1 and RX2 are defined based on Table 7, which corresponds to the duration of five preamble symbols, required to effectively detect the downlink packet preamble. The other parameters used to characterize the consumption in the different phases have been taken from [26,27]; they refer to the Microchip RN2483 LoRa Mote [28], and they are reported in Table 8.

### 5.3. Performance Metrics: Downlink Response Rate

The simulator allows also to estimate the rate of correctly delivered downlink packets. In particular, a downlink packet can be generated for each uplink packet sent by an ED with a user-defined probability. The simulator schedules the downlink packets for each ED based on the number of GWs which have received this packet and their availability (accounting for the DC restrictions). The downlink response rate is then computed as the ratio between the number of downlink packets delivered and those generated.

When modeling the delivery of a downlink, a procedure similar to that discussed above in the case of an uplink is used. However, there are some differences between the RX1 and RX2 cases. According to the LoRaWAN specification, in the case of RX1, data are sent in the same frequency channel used for the uplink, therefore both downlink-to-downlink (i.e., caused by simultaneous downlink transmission from the other GW) and uplink-to-downlink (i.e., caused by simultaneous uplink transmissions of the other ED) are considered. Meanwhile, in the case of RX2, which is typically carried within a standalone frequency channel, only the downlink-to-downlink interference between different GWs is considered.

## 6. Validation

To check the accuracy of the simulator results, several tests have been carried out (results are averaged over 10,000 iteration cycles), and their results have been mainly checked against that of the analytical models and the state-of-the-art literature, as summarized in this section. Comparisons with experimental results have been also carried out, as discussed in the following.

### 6.1. PHY-Layer Simulator

The behavior of the PHY-layer simulator has been validated through the comparison with the results reported in [29,30]. The symbol error rate (i.e., the chirp error rate) as a function of the $\mathrm{SNR}=\frac{P_{u}}{P_{w}}$, where

**Figure 4:**
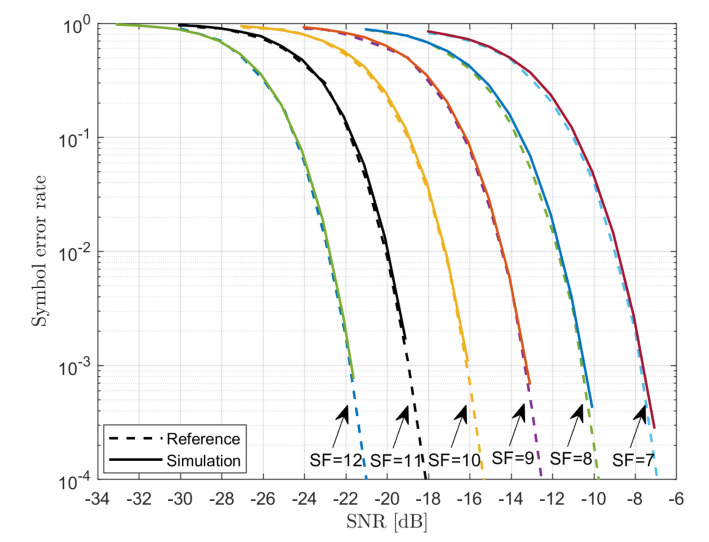
Symbol error rate vs. SNR.

- $P_{u}$ denotes the average power of the signal;
- $P_{w}=N_{0}BW$ denotes the noise power within the nominal signal bandwidth BW.

are presented in Figure 4. Even the visual comparison shows a very close match between the two.

### 6.2. Packet Delivery Rate

The default set of the parameters adopted in our simulations is summarized in Table 9. Note that *T* there stands for the average period between the uplink packets for each ED. As for the scenario, we considered a GW placed at the center of a circular area of radius R and the EDs were randomly, and uniformly distributed within this area.

**Figure 5:**
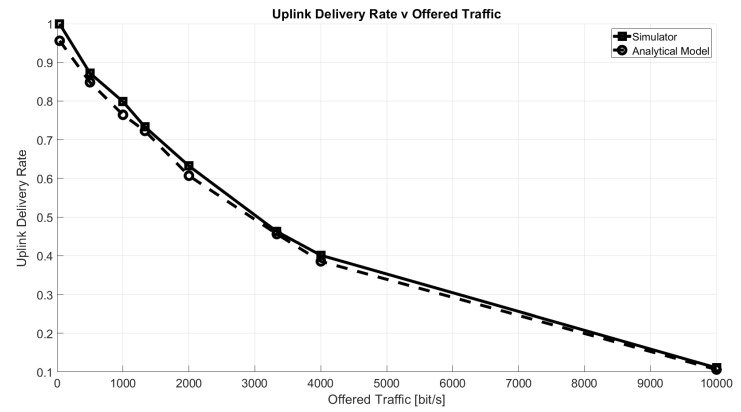
Uplink delivery rate as a function of offered traffic, O.

Figure 5 shows the uplink delivery rate as a function of the offered traffic, defined as:

(12) $$O=\frac{8\cdot B\cdot N}{T}\;\left[\mathrm{bit}/s\right]\;$$

The uplink delivery rate has been defined as the percentage of packets correctly received by the GW according to the conditions described in Section 5.1. As a reference, the figure shows the rate computed with the well-known equation for ALOHA success probability, $P_{s}$, by Abramson [31]:

(13) $$P_{s}=e^{-\frac{2*N*B}{T}}$$

It can be seen that the two curves match very closely. Note that, since the analytic equation accounts only for intra-spreading factor interference and it implies the loss of all collided packets, we disabled, specifically for this comparison, the capture effect and the inter-SF interference computation.

Besides validating the simulator by means of analytical models, an experiment has been carried out by setting up a small LoRaWAN network. To this purpose, we used devices (Idesio Rigers Board 1.0) equipped with the *Microchip RN2483* radio transceiver, fully certified 433/868 MHz SX1276 LoRa module, which supports LoRaWAN Class A, and one gateway, placed 50 m away from the devices. Five boards have been programmed to send a packet of $B_{UL}=16$ bytes every T=60 seconds for 1 h. The uplink delivery rate has been estimated as the ratio between the number of packets received over the number of the sent packet. All devices used the same SF and the experiment has been replicated with SF = 7 and SF = 10. The results reported in Table 10 show the good accuracy of the simulator. Note that the minor difference in the results (i.e., the additional packet losses) may have been caused by an interference from the third-party systems.

### 6.3. Energy Consumption

**Figure 6:**
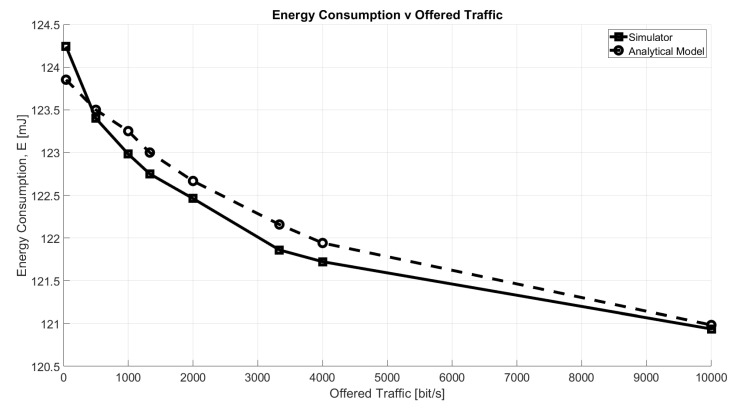
Energy consumption as a function of offered traffic, O.

The validation of the energy consumption results provided by the simulator, has been carried out configuring the GW to respond in RX1 to each packet received in uplink and removing the DC restrictions. The results are presented in Figure 6. For comparison we have used a simple analytical model given by

(14) $$E=E_{UL}+E_{RX_{Delay1}}+E_{DL_{full}}\cdot P_{s}+E_{DL_{empty}}\cdot \left(1-P_{s}\right)\;\left[J\right]$$

where $E_{UL}$ is the energy required for the uplink transmission; $E_{RX_{Delay1}}$ is the energy consumed between the uplink and RX1 (having duration of 1 s); in downlink, $E_{DL_{full}}$ is the energy spent if a packet is received; and finally, $E_{DL_{empty}}$ denotes the consumption of a device when it opens the RX Window, but no packet is received. The NS will send a packet in downlink only if it receives an uplink packet, whose success probability is equal to $P_{s}$, so it knows the device has transmitted something and it will open the receive window. It should be highlighted that in this case, we do not take into account duty cycle constraints or the possibility of a downlink message being sent in RX2, which would require a more complex model. As can be seen from the presented charts, the results are almost coincident.

## 7. Numerical Results

Having validated the simulator in simple scenarios, in this section, we proceed by delivering some initial insight into less trivial cases, which show some notable trade-offs. Unless stated otherwise, for these simulations, we use the same parameters and configurations, which have been used in the previous section. In all the considered scenarios, GWs operate in full-duplex mode.

**Figure 7:**
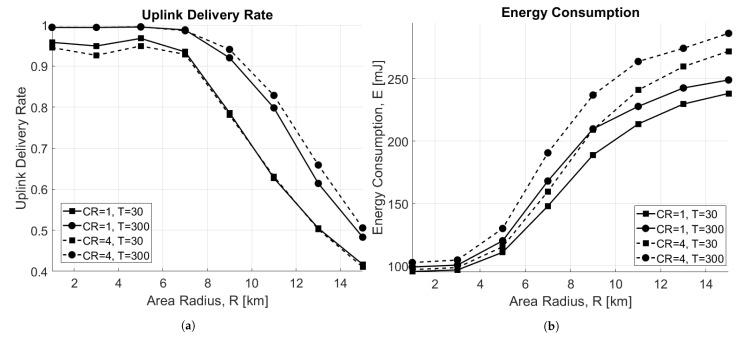
Impact of the area radius, R (km). (**a**) Uplink delivery rate as a function of area radius, R. (**b**) Energy consumption as a function of area radius, R.

Figure 7a depicts the uplink delivery rate as a function of the radius of the circular area where N=50 nodes are distributed for both CR=1 and CR=4, when varying *T*. As expected, when the area dimension increases, the delivery rate decreases. In particular, in an interference-limited scenario (T=30 s), performance does not depend on CR, but for very small areas, CR=1 is the best case due to lower ToA, and therefore, collisions. On the other hand, when the scenario is limited by noise (T=300 s), CR=4 performs better for bigger areas, thanks to the stronger encoding it is able to offer.

Observing the energy performance in this scenario, reported in Figure 7b, it can be noticed that the consumption increases with the area size. This happens because EDs are forced to use higher SFs when they are far from the GW, which causes higher energy consumption. In addition, nodes using CR=4 consume more energy mainly because the ToA is higher; therefore, more time is spent in transmission.

**Figure 8:**
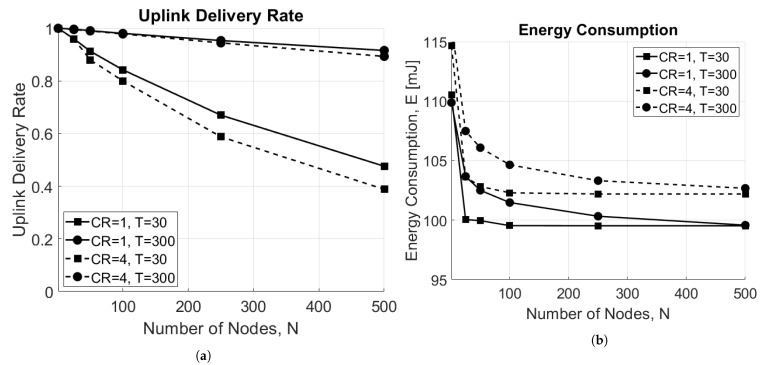
Impact of the number of end devices, N. (**a**) Uplink delivery rate as a function of number of EDs, N. (**b**) Energy consumption as a function of number of EDs, N.

Figure 8a shows the delivery rate as a function of the number of EDs in the network for both CR=1 and CR=4. As a matter of fact, the higher is the number of EDs in the network, the higher is the rate of collisions, which results in the degradation of the overall performance of the network. In addition, when the scenario is limited by interference (T=30 s), CR=1 shows better performance. This happens because the ToA is smaller with respect to CR=4 case; therefore, there are fewer collisions. On the other hand, when the scenario is limited by noise (T=300 s) and all EDs can successfully reach the GW since no interference is present, no difference between the two approaches are highlighted. The respective energy consumption is revealed in Figure 8b, which shows that the average energy consumption decreases when the number of ED increases. This result may seem counter-intuitive; however, it is explained by the increase of the uplink collisions, resulting in fewer packets being correctly received by the GW, and consequently, fewer packets sent in downlink to the ED. As a matter of fact, if the uplink message is lost, no downlink packet will be sent to the ED, which will then consume less power. In addition, also in this case, nodes using CR=4 consume more energy on average.

For the sake of completeness, an analysis of the uplink response rate in presence of class C downlink traffic has been carried out, whose functioning has been described in Section 3.2. In this scenario, N=50 EDs transmit every T=60 s. G=2 GWs have been placed at (x,y)=[R/2,R/2],[−R/2,−R/2] of a circular area of radius *R*. More specifically, we assumed that one GW may be transmitting a class C downlink packet with probability *p* at an instant t∈[0,T], and therefore, it may be interfering with the uplink packet sent to the other GW, according to the capture effect mechanism already described in Section 5.1. As expected, the higher the probability *p*, the higher the interference, so the uplink response rate decreases. Results are provided in Table 11.

**Figure 9:**
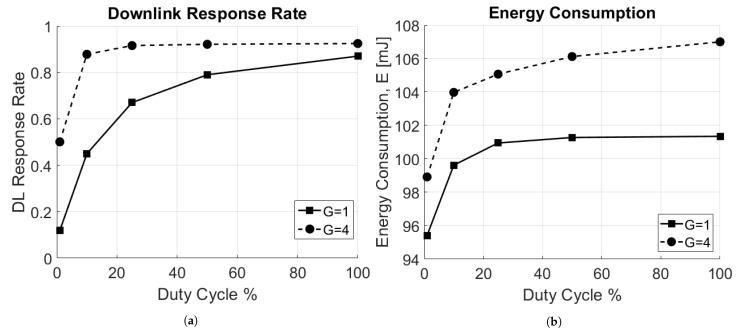
Impact of the duty cycle, DC. (**a**) Downlink response rate. (**b**) Energy consumption.

Finally, an analysis of the downlink response rate, as described in Section 5.3, has been carried out. Two scenarios have been considered: one with a single GW, placed at the center of the circular area of radius *R*, and one with four GWs, placed respectively at coordinates (x,y)=[R/2,R/2];[−R/2,−R/2];[R/2,−R/2];[−R/2,R/2], with RX2 disabled. In Figure 9a the downlink response rate as a function of the duty cycle for RX1 window is reported. The higher the DC, the higher is the number of packets sent in the downlink. Per intuition, with more GWs present in the network, the downlink response rate is higher. However, this results in increased average energy consumption, since the reception of a downlink packet often requires more energy than that needed for checking the two empty receive windows.

## 8. Conclusions

With the number of LoRaWAN connections approaching 200 million worldwide [32], there is significant interest from both scientific and industrial communities in this technology. However, despite the apparent simplicity of the LoRaWAN protocol, assessing its performance and finding the proper trade-offs by means of analytical tools is not always possible. Therefore, in this paper, we present and openly deliver to the community a new MATLAB-based simulator tool covering the physical and the upper layers of the LoRa/LoRaWAN protocol stack. As we demonstrate in the paper, the proposed simulator has a number of the unique features (including, but not limited to (i) support of multiple gateways both for uplink and downlink; (ii) accounting for uplink–uplink, uplink–downlink, downlink–uplink and downlink–downlink interference; (iii) receiving window prioritization; (iv) modeling of energy consumption) whilst being decently simple to use and easy to configure. Importantly, it is built using MATLAB, which is already familiar to many scholars in the field and thus has a less steep learning curve than many other LoRaWAN simulators.

Besides, we have presented some illustrative results obtained using the developed simulator, which demonstrate exciting and not always intuitive trade-offs. First, we investigated the impacts of different coding rates in interference-limited and noise-limited scenarios, showing that some benefit on the packet delivery rate, although marginal, can be achieved only in the latter scenario. Notably, we showed that such benefit comes at the price of an increased energy consumption. Second, we showed that as the number of EDs increases significantly, thereby making the network operate in heavily interference limited conditions, the adoption of more powerful coding rates is counterproductive, for both the delivery rate and the energy consumption. Third, we assessed the impact of downlink transmissions (e.g., acknowledgments) on the average energy consumption of EDs, also showing that increasing the number of GWs affects not only the packet delivery rates in uplink and downlink but also the average consumption of devices, and hence, ultimately, the battery life.

All these and many other aspects of LoRaWAN require further investigations, and we believe that the simulator presented in this paper can be a great help. In the future, we plan to extend the simulator with new functionalities missing as of today, such as more advanced propagation models, enabling the support of multiple channels and other device classes and providing a more dynamic formation of data traffic.

## Figures and Tables

**Table 1:** Bit rate with BW = 125 kHz and CR = 1.

SF	7	8	9	10	11	12
Bit rate [bit/s]	5468	3125	1757	976	537	293

**Table 2:** EU868 default channels.

Channel Frequency [MHz]	868.1	868.3	868.5
BW [kHz]	125		

**Table 3:** $SNR_{\min}$ values for different SF with BW = 125 kHz [23].

SF	7	8	9	10	11	12
$SNR_{\min}$ [dB]	−20	−17.5	−15	−12.5	−10	−7.5

**Table 4:** RX1DROffset [10].

	RX1DROffset	0	1	2	3	4	5
Uplink SF							
7		7	8	9	10	11	12
8		8	9	10	11	12	12
9		9	10	11	12	12	12
10		10	11	12	12	12	12
11		11	12	12	12	12	12
12		12	12	12	12	12	12

**Table 5:** List of parameters defined by the user.

Network Layout Parameters			
*N*	Number of EDs	*G*	Number of GWs
*R*	Circular area radius [km]		
**Radio propagation parameters**			
$P_{T}^{ED}$	Transmit power of the ED [dBm]	$P_{T}^{GW}$	Transmit power of the GW [dBm]
$G_{A}^{ED}$	ED Antenna Gain [dB]	$G_{A}^{GW}$	GW Antenna Gain [dB]
$h_{m}$	Height of the ED [m]	$h_{b}$	Height of the GW [m]
σ	Shadowing standard deviation [dB]		
**Radio resource management parameters**			
SF	Spreading Factor	RX1DROffset	Shift between Uplink and RX1 SF
DC	Duty Cycle Limitation		
**Data traffic parameters**			
$B_{UL}$	Uplink Payload Size [bytes]	$B_{DL}$	Downlink Payload Size [bytes]
*H*	Packet Header presence	$L_{preamble}$	Length of the preamble
*T*	Uplink packet periodicity [s]		
**SIR threshold parameters**			
γ	SIR Threshold [dB]		
**Physical layer parameters**			
CR	Coding Rate	BW	Sweep interval [kHz]

**Table 6:** SIR Thresholds [dB] [8].

	Int	SF7	SF8	SF9	SF10	SF11	SF12
Ref							
SF7		1	−8	−9	−9	−9	−9
SF8		−11	1	−11	−12	−13	−13
SF9		−15	−13	1	−13	−14	−15
SF10		−19	−18	−17	1	−17	−18
SF11		−22	−22	−21	−11	1	−20
SF12		−25	−25	−25	−24	−23	1

**Table 7:** RX1 and RX2 duration when no packets are received by the ED [26].

SF	7	8	9	10	11	12
$T_{RX1}$ [s]	12.29	24.58	49.14	98.3	131.02	262.14
$T_{RX2}$ [s]	1.28	2.3	4.35	8.45	16.64	33.02

**Table 8:** Energy Consumption parameters [27].

$P_{T}$ [dBm]	14	12	10	8	6	4	2
$I_{TX}$ [mA]	38	35.1	32.4	30	27.5	24.7	22.3
$I_{RX}$ [mA]	38						
$I_{RX_{Delay}}$ [mA]	27						
$I_{Sleep}$ [mA]	0.0016						
*V* [V]	3.3						

**Table 9:** Simulation parameters.

*N*	{1, 25, 50, 100, 250, 500}	$P_{T}^{ED}$	14 [dBm]
*G*	1	$P_{T}^{GW}$	16 [dBm]
$G_{A}^{ED}$	0 [dB]	$G_{A}^{GW}$	0 [dB]
$B_{UL}$	20 [bytes]	$B_{DL}$	20 [bytes]
*R*	4 [km]	CR	1
*T*	{10, 30, 60, 150, 300} [s]	BW	125 [kHz]
$h_{m}$	1 [m]	$h_{b}$	30 [m]
*H*	1	$L_{preamble}$	8
σ	3 [dB]	RX1DROffset	0
DC	1%		

**Table 10:** Experimental results and comparison with analytical, simulated and experimental results.

Results	SF = 7	SF = 10
Analytical Result	0.991	0.959
Experimental Result	0.968	0.914
Simulated Result	0.997	0.978

**Table 11:** Uplink response rate with Class C downlink interference.

p	Uplink Delivery Rate
0	0.9748
0.1	0.9662
0.3	0.9428
0.5	0.894
